# Common Coupled Fixed Point Theorems for Two Hybrid Pairs of Mappings under *φ*-*ψ* Contraction

**DOI:** 10.1155/2014/608725

**Published:** 2014-11-27

**Authors:** Bhavana Deshpande, Amrish Handa

**Affiliations:** Department of Mathematics, Govt. PG Arts & Science College, Ratlam, Madhya Pradesh 457001, India

## Abstract

We introduce the concept of (EA) property and occasional *w*-compatibility for hybrid pair *F* : *X* × *X* → 2^*X*^ and *f* : *X* → *X*. We also introduce common (EA) property for two hybrid pairs *F*, *G* : *X* → 2^*X*^ and *f*, *g* : *X* → *X*. We establish some common coupled fixed point theorems for two hybrid pairs of mappings under *φ*-*ψ* contraction on noncomplete metric spaces. An example is also given to validate our results. We improve, extend and generalize several known results. The results of this paper generalize the common fixed point theorems for hybrid pairs of mappings and essentially contain fixed point theorems for hybrid pair of mappings.

## 1. Introduction and Preliminaries

Let (*X*, *d*) be a metric space and let CB(*X*) be the set of all nonempty closed bounded subsets of *X*. Let *D*(*x*, *A*) denote the distance from *x* to *A* ⊂ *X* and let *H* denote the Hausdorff metric induced by *d*; that is,

(1) Dx,A=inf⁡a∈Adx,a,HA,B=max⁡sup⁡a∈ADa,B,sup⁡b∈BDb,A,∀A,B∈CBX.

The study of fixed points for multivalued contractions and nonexpansive mappings using the Hausdorff metric was initiated by Markin [1]. The existence of fixed points for various multivalued contractive mappings has been studied by many authors under different conditions. The theory of multivalued mappings has application in control theory, convex optimization, differential inclusions, and economics. In 1969, Nadler [2] extended the famous Banach contraction principle [3] from single-valued mapping to multivalued mapping and proved the fixed point theorem for the multivalued contraction. Many authors proved fixed point theorems for hybrid pair of mappings without assuming the continuity of any mapping involved including [4–7].

In [8], Gnana Bhaskar and Lakshmikantham established some coupled fixed point theorems and applied these results to study the existence and uniqueness of solution for periodic boundary value problems. Luong and Thuan [9] generalized the results of Gnana Bhaskar and Lakshmikantham [8]. Berinde [10] extended the results of Gnana Bhaskar and Lakshmikantham [8] and Luong and Thuan [9]. Lakshmikantham and Ćirić [11] proved coupled coincidence and common coupled fixed point theorems for nonlinear contractive mappings in partially ordered complete metric spaces and extended the results of Gnana Bhaskar and Lakshmikantham [8]. Jain et al. [12] extended and generalized the results of Berinde [10], Gnana Bhaskar and Lakshmikantham [8], Lakshmikantham and Ćirić [11], and Luong and Thuan [9].

Deshpande and Handa [13] generalized and intuitionistically fuzzified the results of Gnana Bhaskar and Lakshmikantham [8], Lakshmikantham and Ćirić [11], and Luong and Thuan [9], while Deshpande et al. [14] generalized and intuitionistically fuzzified the results of Berinde [10], Gnana Bhaskar and Lakshmikantham [8], Lakshmikantham and Ćirić [11], and Luong and Thuan [9]. In [15], Deshpande et al. proved a common coupled fixed point theorem for mappings under *φ*-contractive conditions on intuitionistic fuzzy metric spaces. As an application, the existence and uniqueness of solution to a nonlinear Fredholm integral equation have been studied.

Recently Samet et al. [16] claimed that most of the coupled fixed point theorems in the setting of single valued mappings on ordered metric spaces are consequences of well-known fixed point theorems.

These concepts were extended by Abbas et al. [17] to multivalued mappings and who obtained coupled coincidence point and common coupled fixed point theorems involving hybrid pair of mappings satisfying generalized contractive conditions in complete metric spaces. Very few authors studied coupled fixed point theorems for hybrid pair of mappings including [17–20].

In [17], Abbas et al. introduced the following concept.

**Definition 1 .**

Let *X* be a nonempty set, *F* : *X* × *X* → 2^*X*^ (a collection of all nonempty subsets of *X*), and let *g* be a self-mapping on *X*. An element (*x*, *y*) ∈ *X* × *X* is called

(1) a coupled coincidence point of hybrid pair {*F*, *g*} if *g*(*x*) ∈ *F*(*x*, *y*) and *g*(*y*) ∈ *F*(*y*, *x*),
(2) a common coupled fixed point of hybrid pair {*F*, *g*} if *x* = *g*(*x*) ∈ *F*(*x*, *y*) and *y* = *g*(*y*) ∈ *F*(*y*, *x*).

We denote the set of coupled coincidence points of mappings *F* and *g* by *C*{*F*,*g*}. Note that if (*x*, *y*) ∈ *C*{*F*, *g*}, then (*y*, *x*) is also in *C*{*F*, *g*}.

**Definition 2 .**

Let *F* : *X* × *X* → 2^*X*^ be a multivalued mapping and let *g* be a self-mapping on *X*. The hybrid pair {*F*, *g*} is called *w*-compatible if *g*(*F*(*x*, *y*))⊆*F*(*gx*, *gy*) whenever (*x*, *y*) ∈ *C*{*F*, *g*}.

**Definition 3 .**

Let *F* : *X* × *X* → 2^*X*^ be a multivalued mapping and let *g* be a self-mapping on *X*. The mapping *g* is called *F*-weakly commuting at some point (*x*, *y*) ∈ *X* × *X* if *g* ^2^ *x* ∈ *F*(*gx*, *gy*) and *g* ^2^ *y* ∈ *F*(*gy*, *gx*).

Aamri and El Moutawakil [21] defined (EA) property for self-mappings which contained the class of noncompatible mappings. Kamran [22] extended the (EA) property for hybrid pair *f* : *X* → *X* and *T* : *X* → 2^*X*^. Liu et al. [23] introduced common (EA) property for hybrid pairs of single and multivalued mappings and gave some new common fixed point theorems under hybrid contractive conditions. Abbas and Rhoades [24] extended the concept of occasionally weakly compatible mappings for hybrid pair *f* : *X* → *X* and *T* : *X* → 2^*X*^.

In this paper, we introduce the concept of (EA) property and occasional *w*-compatibility for hybrid pair *F* : *X* × *X* → 2^*X*^ and *f* : *X* → *X*. We also introduce common (EA) property for two hybrid pairs *F*, *G* : *X* × *X* → 2^*X*^ and *f*, *g* : *X* → *X*. We establish some common coupled fixed point theorems for two hybrid pairs of mappings under *φ*-*ψ* contraction on noncomplete metric spaces. The *φ*-*ψ* contraction is weaker contraction than the contraction defined in Gnana Bhaskar and Lakshmikantham [8] and Luong and Thuan [9]. We improve, extend, and generalize the results of Berinde [10], Gnana Bhaskar and Lakshmikantham [8], Jain et al. [12], Lakshmikantham and Ćirić [11], Liu et al. [23], and Luong and Thuan [9]. The results of this paper generalize the common fixed point theorems for hybrid pairs of mappings and essentially contain fixed point theorems for hybrid pair of mappings.

## 2. Main Results

We first define the following.

**Definition 4 .**

Mappings *f* : *X* → *X* and *F* : *X* × *X* → CB(*X*) are said to satisfy the (EA) property if there exist sequences {*x*_*n*_}, {*y*_*n*_} in *X*, some *u*, *v* in *X*, and *A*,  *B* in CB(*X*) such that

(2) $$\begin{array}{c} \underset{n\to \infty }{\lim}fx_{n}=u\in A=\underset{n\to \infty }{\lim}F\left(x_{n},y_{n}\right),\\ \underset{n\to \infty }{\lim}fy_{n}=v\in B=\underset{n\to \infty }{\lim}F\left(y_{n},x_{n}\right). \end{array}$$

**Definition 5 .**

Let *f*, *g* : *X* → *X* and *F*, *G* : *X* × *X* → CB(*X*). The pairs {*F*, *f*} and {*G*, *g*} are said to satisfy the common (EA) property if there exist sequences {*x*_*n*_}, {*y*_*n*_}, {*u*_*n*_}, and {*v*_*n*_} in *X*, some *u*, *v* in *X*, and *A*, *B*, *C*, *D* in CB(*X*) such that

(3) $$\begin{array}{c} \underset{n\to \infty }{\lim}F\left(x_{n},y_{n}\right)=A,\;\;\underset{n\to \infty }{\lim}G\left(u_{n},v_{n}\right)=B,\\ \underset{n\to \infty }{\lim}fx_{n}=\underset{n\to \infty }{\lim}gu_{n}=u\in A\cap B,\\ \underset{n\to \infty }{\lim}F\left(y_{n},x_{n}\right)=C,\;\;\underset{n\to \infty }{\lim}G\left(v_{n},u_{n}\right)=D,\\ \underset{n\to \infty }{\lim}fy_{n}=\underset{n\to \infty }{\lim}gv_{n}=v\in C\cap D. \end{array}$$

**Example 6 .**

Let *X* = [1, +*∞*) with the usual metric. Define *f*, *g* : *X* → *X* and *F*, *G* : *X* × *X* → CB(*X*) by

(4) $$\begin{array}{c} F\left(x,y\right)=\left[2,3+2x+y\right],\;\;G\left(x,y\right)=\left[2,3+\frac{3x+y}{4}\right]\\ f\left(x\right)=2+x,\;g\left(x\right)=1+\frac{x}{2},\;\forall x,y\in X. \end{array}$$

Consider the sequences

(5) $$\begin{array}{c} \left\{x_{n}\right\}=\left\{2+\frac{1}{n}\right\},\;\;\left\{y_{n}\right\}=\left\{4+\frac{1}{n}\right\},\\ \left\{u_{n}\right\}=\left\{6+\frac{1}{n}\right\},\;\;\left\{v_{n}\right\}=\left\{10+\frac{1}{n}\right\}. \end{array}$$

Clearly,

(6) $$\begin{array}{c} \underset{n\to \infty }{\lim}F\left(x_{n},y_{n}\right)=\left[2,11\right]=A,\\ \underset{n\to \infty }{\lim}G\left(u_{n},v_{n}\right)=\left[2,10\right]=B,\\ \underset{n\to \infty }{\lim}fx_{n}=\underset{n\to \infty }{\lim}gu_{n}=4\in A\cap B,\\ \underset{n\to \infty }{\lim}F\left(y_{n},x_{n}\right)=\left[2,13\right]=C,\\ \underset{n\to \infty }{\lim}G\left(v_{n},u_{n}\right)=\left[2,12\right]=D,\\ \underset{n\to \infty }{\lim}fy_{n}=\underset{n\to \infty }{\lim}gv_{n}=6\in C\cap D. \end{array}$$

Therefore, the pairs {*F*, *f*} and {*G*, *g*} are said to satisfy the common (EA) property.

**Definition 7 .**

Mappings *F* : *X* × *X* → 2^*X*^ and *f* : *X* → *X* are said to be occasionally *w*-compatible if and only if there exists some point (*x*, *y*) ∈ *X* × *X* such that *fx* ∈ *F*(*x*, *y*), *fy* ∈ *F*(*y*, *x*), and *fF*(*x*, *y*)⊆*F*(*fx*, *fy*).

**Example 8 .**

Let *X* = [0, +*∞*) with usual metric. Define *f* : *X* → *X*, *F* : *X* × *X* → CB(*X*) by

(7) $$\begin{array}{c} fx=\left\{\begin{array}{cc} 0, & 0\leq x<1,\\ 4x, & 1\leq x<\infty , \end{array}\right.\\ F\left(x,y\right)=\left\{\begin{array}{cc} \left[0,1+2x+y\right], & \left(x,y\right)\neq \left(0,0\right),\\ \left\{x\right\}, & \left(x,y\right)=\left(0,0\right). \end{array}\right. \end{array}$$

It can be easily verified that (0,0) and (1,1) are coupled coincidence points of *f* and *F*, but *fF*(0,0)⊆*F*(*f*0, *f*0) and fF(1,1)⫅F(f1,f1). So *f* and *F* are not *w*-compatible. However, the pair {*F*, *f*} is occasionally *w*-compatible.

Let Φ denote the set of all functions *φ* : [0, +*∞*)→[0, +*∞*) satisfying the following:

 (i_*φ*_)  *φ* is continuous and strictly increasing,
 (ii_*φ*_)  *φ*(*t*) < *t* for all *t* > 0,
 (iii_*φ*_)  *φ*(*t* + *s*) ≤ *φ*(*t*) + *φ*(*s*) for all *t*, *s* > 0.

And let Ψ denote the set of all functions *ψ* : [0, +*∞*)→[0, +*∞*) which satisfies

 (i_*ψ*_)  lim⁡_*t*→*r*_ *ψ*(*t*) > 0 for all *r* > 0 and lim⁡_*t*→0+_ *ψ*(*t*) = 0,
 (ii_*ψ*_)  *ψ*(*t*) > 0 for all *t* > 0 and *ψ*(0) = 0.

Note that, by (i_*φ*_) and (ii_*φ*_), we have that *φ*(*t*) = 0 if and only if *t* = 0. For example, functions *φ*_1_(*t*) = *kt* where *k* > 0, *φ*_2_(*t*) = *t*/(*t* + 1), *φ*_3_(*t*) = ln⁡(*t* + 1), and *φ*_4_(*t*) = min⁡{*t*, 1} are in Φ,  *ψ*_1_(*t*) = *kt* where *k* > 0,  *ψ*_2_(*t*) = (ln⁡(2*t* + 1))/2, and $\psi_{3}(t)=\left\{\begin{array}{cc} 1, & t=0\\ t/\left(t+1\right), & 0<t<1\\ 1, & t=1\\ t/2, & t>1 \end{array}\right.$ are in Ψ.

Now, we prove our main results.

**Theorem 9 .**

Let (*X*, *d*) be a metric space. Assume *F*, *G* : *X* × *X* → CB(*X*) and *f*, *g* : *X* → *X* to be mappings satisfying the following.

(1) {*F*, *f*} and {*G*, *g*} satisfy the common (EA) property.
(2) For all *x*, *y*, *u*, *v* ∈ *X*, there exist some *φ* ∈ Φ and some *ψ* ∈ Ψ such that
  (8) φHFx,y,Gu,v+HFy,x,Gv,u2 ≤φdfx,gu+dfy,gv2  −ψdfx,gu+dfy,gv2.
(3) *f*(*X*) and *g*(*X*) are closed subsets of *X*. Then
  (a) *F* and *f* have a coupled coincidence point,
  (b) *G* and *g* have a coupled coincidence point,
  (c) *F* and *f* have a common coupled fixed point, if *f* is *F*-weakly commuting at (*x*, *y*) and *f* ^2^ *x* = *fx* and *f* ^2^ *y* = *fy* for (*x*, *y*) ∈ *C*{*F*, *f*},
  (d) *G* and *g* have a common coupled fixed point, if *g* is *G*-weakly commuting at $\left(\tilde{x},\tilde{y}\right)$ and $g^{2}\tilde{x}=g\tilde{x}$ and $g^{2}\tilde{y}=g\tilde{y}$ for $\left(\tilde{x},\tilde{y})\in C\{G,g\right\}$,
  (e) *F*, *G*, *f*,  and *g* have common coupled fixed point provided that both (c) and (d) are true.

**Proof**

Since {*F*, *f*} and {*G*, *g*} satisfy the common (EA) property, there exist sequences {*x*_*n*_}, {*y*_*n*_}, {*u*_*n*_}, and {*v*_*n*_} in *X*, some *u*, *v* in *X*, and *A*, *B*, *C*, *D* in CB(*X*) such that

(9) $$\begin{array}{c} \underset{n\to \infty }{\lim}F\left(x_{n},y_{n}\right)=A,\;\;\underset{n\to \infty }{\lim}G\left(u_{n},v_{n}\right)=B,\\ \underset{n\to \infty }{\lim}fx_{n}=\underset{n\to \infty }{\lim}gu_{n}=u\in A\cap B,\\ \underset{n\to \infty }{\lim}F\left(y_{n},x_{n}\right)=C,\;\;\underset{n\to \infty }{\lim}G\left(v_{n},u_{n}\right)=D,\\ \underset{n\to \infty }{\lim}fy_{n}=\underset{n\to \infty }{\lim}gv_{n}=v\in C\cap D. \end{array}$$

Since *f*(*X*) and *g*(*X*) are closed subsets of *X*, then there exist $x,y,\tilde{x},\tilde{y}\in X$,

(10) $$\begin{array}{c} u=fx=g\tilde{x},\;\;v=fy=g\tilde{y}. \end{array}$$

Now, by using condition (2) of Theorem 9, we get

(11) φHFx,y,Gun,vn+HFy,x,Gvn,un2 ≤φdfx,gun+dfy,gvn2  −ψdfx,gun+dfy,gvn2.

Letting *n* → *∞* in the above inequality, by using (9), (10), (i_*φ*_),  (ii_*φ*_), and (i_*ψ*_), we obtain

(12) φHFx,y,B+HFy,x,D2  ≤φ0−0=0−0=0,

which, by (i_*φ*_) and (ii_*φ*_), implies

(13) $$\begin{array}{c} H\left(F\left(x,y\right),B\right)=0,\;\;H\left(F\left(y,x\right),D\right)=0. \end{array}$$

Since *fx* ∈ *B* and *fy* ∈ *D*, it follows that

(14) $$\begin{array}{c} fx\in F\left(x,y\right),\;\;fy\in F\left(y,x\right). \end{array}$$

That is, (*x*, *y*) is a coupled coincidence point of *F* and *f*. This proves (a). Again, by using condition (2) of Theorem 9, we get

(15) φHFxn,yn,Gx~,y~+HFyn,xn,Gy~,x~2 ≤φdfxn,gx~+dfyn,gy~2  −ψdfxn,gx~+dfyn,gy~2.

Letting *n* → *∞* in the above inequality, by using (9), (10), (i_*φ*_),  (ii_*φ*_), and (i_*ψ*_), we obtain

(16) φHA,Gx~,y~+HC,Gy~,x~2  ≤φ0−0=0−0=0,

which, by (i_*φ*_) and (ii_*φ*_), implies

(17) $$\begin{array}{c} H\left(A,G\left(\tilde{x},\tilde{y}\right)\right)=0,\;\;H\left(C,G\left(\tilde{y},\tilde{x}\right)\right)=0. \end{array}$$

Since $g\tilde{x}\in A$ and $g\tilde{y}\in C$, it follows that

(18) $$\begin{array}{c} g\tilde{x}\in G\left(\tilde{x},\tilde{y}\right),\;\;g\tilde{y}\in G\left(\tilde{y},\tilde{x}\right). \end{array}$$

That is, $\left(\tilde{x},\tilde{y}\right)$ is a coupled coincidence point of *G* and *g*. This proves (b).

Furthermore, from condition (c), we have *f* which is *F*-weakly commuting at (*x*, *y*); that is, *f* ^2^ *x* ∈ *F*(*fx*, *fy*), *f* ^2^ *y* ∈ *F*(*fy*, *fx*) and *f* ^2^ *x* = *fx*, *f* ^2^ *y* = *fy*. Thus, *fx* = *f* ^2^ *x* ∈ *F*(*fx*, *fy*) and *fy* = *f* ^2^ *y* ∈ *F*(*fy*, *fx*); that is, *u* = *fu* ∈ *F*(*u*, *v*) and *v* = *fv* ∈ *F*(*v*, *u*). This proves (c). A similar argument proves (d). Then (e) holds immediately.

Put *f* = *g* in Theorem 9, and we get the following result.

**Corollary 10 .**

Let (*X*, *d*) be a metric space. Assume *F*, *G* : *X* × *X* → CB(*X*) and *g* : *X* → *X* to be mappings such that

(1) {*F*, *g*} and {*G*, *g*} satisfy the common (EA) property,
(2) for all *x*,*y*,*u*,*v* ∈ *X*, there exist some *φ* ∈ Φ and some *ψ* ∈ Ψ such that
  (19) φHFx,y,Gu,v+HFy,x,Gv,u2 ≤φdgx,gu+dgy,gv2  −ψdgx,gu+dgy,gv2,
(3) *g*(*X*) is a closed subset of *X*. Then
  (a) *F* and *g* have a coupled coincidence point,
  (b) *G* and *g* have a coupled coincidence point,
  (c) *F* and *g* have a common coupled fixed point, if *g* is *F*-weakly commuting at (*x*, *y*) and *g* ^2^ *x* = *gx* and *g* ^2^ *y* = *gy* for (*x*, *y*) ∈ *C*{*F*, *g*},
  (d) *G* and *g* have a common coupled fixed point, if *g* is *G*-weakly commuting at $\left(\tilde{x},\tilde{y}\right)$ and $g^{2}\tilde{x}=g\tilde{x}$ and $g^{2}\tilde{y}=g\tilde{y}$ for $\left(\tilde{x},\tilde{y})\in C\{G,g\right\}$,
  (e) *F*, *G*,  and *g* have common coupled fixed point provided that both (c) and (d) are true.

Put *F* = *G* and *f* = *g* in Theorem 9, and we get the following result.

**Corollary 11 .**

Let (*X*, *d*) be a metric space. Assume *F* : *X* × *X* → CB(*X*) and *g* : *X* → *X* to be mappings such that

(1) {*F*, *g*} satisfies the (EA) property,
(2) for all *x*, *y*, *u*, *v* ∈ *X*, there exist some *φ* ∈ Φ and some *ψ* ∈ Ψ such that
  (20) φHFx,y,Fu,v+HFy,x,Fv,u2 ≤φdgx,gu+dgy,gv2  −ψdgx,gu+dgy,gv2.

If (3) of Corollary 10 holds. Then

(a) *F* and *g* have a coupled coincidence point,
(b) *F* and *g* have a common coupled fixed point, if *g* is *F*-weakly commuting at (*x*, *y*) and *g* ^2^ *x* = *gx* and *g* ^2^ *y* = *gy* for (*x*, *y*) ∈ *C*{*F*, *g*}.

**Corollary 12 .**

Let (*X*, *d*) be a metric space. Assume *F*, *G* : *X* × *X* → CB(*X*) and *f*, *g* : *X* → *X* to be mappings satisfying (1) of Theorem 9 and

(1) for all *x*,*y*,*u*,*v* ∈ *X*, there exists some *ψ* ∈ Ψ such that
  (21) HFx,y,Gu,v+HFy,x,Gv,u ≤dfx,gu+dfy,gv  −2ψdfx,gu+dfy,gv2.

If (3) of Theorem 9 holds, then

(a) *F* and *f* have a coupled coincidence point,
(b) *G* and *g* have a coupled coincidence point,
(c) *F* and *f* have a common coupled fixed point, if *f* is *F*-weakly commuting at (*x*, *y*) and *f* ^2^ *x* = *fx* and *f* ^2^ *y* = *fy* for (*x*, *y*) ∈ *C*{*F*, *f*},
(d) *G* and *g* have a common coupled fixed point, if *g* is *G*-weakly commuting at $\left(\tilde{x},\tilde{y}\right)$ and $g^{2}\tilde{x}=g\tilde{x}$ and $g^{2}\tilde{y}=g\tilde{y}$ for $\left(\tilde{x},\tilde{y})\in C\{G,g\right\}$,
(e) *F*, *G*, *f*, and  *g* have common coupled fixed point provided that both (c) and (d) are true.

**Proof**

If *ψ* ∈ Ψ, then for all *r* > 0, *rψ* ∈ Ψ. Now divide condition (1) of Corollary 12 by 4 and take *φ*(*t*) = (1/2)*t*, *t* ∈ [0, +*∞*), and then the above condition reduces to condition (2) of Theorem 9 with *ψ*_1_ = (1/2)*ψ* and hence by Theorem 9 we get Corollary 12.

Put *f* = *g* in Corollary 12, and we get the following result.

**Corollary 13 .**

Let (*X*, *d*) be a metric space. Assume *F*, *G* : *X* × *X* → CB(*X*) and *g* : *X* → *X* to be mappings satisfying (1) of Corollary 10 and

(1) for all *x*, *y*, *u*, *v* ∈ *X*, there exists some *ψ* ∈ Ψ such that
  (22) HFx,y,Gu,v+HFy,x,Gv,u ≤dgx,gu+dgy,gv  −2ψdgx,gu+dgy,gv2.

If (3) of Corollary 10 holds, then

(a) *F* and *g* have a coupled coincidence point,
(b) *G* and *g* have a coupled coincidence point,
(c) *F* and *g* have a common coupled fixed point, if *g* is *F*-weakly commuting at (*x*, *y*) and *g* ^2^ *x* = *gx* and *g* ^2^ *y* = *gy* for (*x*, *y*) ∈ *C*{*F*, *g*},
(d) *G* and *g* have a common coupled fixed point, if *g* is *G*-weakly commuting at $\left(\tilde{x},\tilde{y}\right)$ and $g^{2}\tilde{x}=g\tilde{x}$ and $g^{2}\tilde{y}=g\tilde{y}$ for $\left(\tilde{x},\tilde{y})\in C\{G,g\right\}$,
(e) *F*, *G*, and  *g* have common coupled fixed point provided that both (c) and (d) are true.

Put *F* = *G* and *f* = *g* in Corollary 12, we get the following result.

**Corollary 14 .**

Let (*X*, *d*) be a metric space. Assume *F* : *X* × *X* → CB(*X*) and *g* : *X* → *X* to be mappings satisfying (1) of Corollary 11 and

(1) for all *x*, *y*, *u*, *v* ∈ *X*, there exists some *ψ* ∈ Ψ such that
  (23) HFx,y,Fu,v+HFy,x,Fv,u ≤dgx,gu+dgy,gv  −2ψdgx,gu+dgy,gv2.

If (3) of Corollary 10 holds, then

(a) *F* and *g* have a coupled coincidence point,
(b) *F* and *g* have a common coupled fixed point, if *g* is *F*-weakly commuting at (*x*, *y*) and *g* ^2^ *x* = *gx* and *g* ^2^ *y* = *gy* for (*x*, *y*) ∈ *C*{*F*, *g*}.

**Theorem 15 .**

Let (*X*, *d*) be a metric space. Assume *F*, *G* : *X* × *X* → CB(*X*) and *f*, *g* : *X* → *X* to be mappings satisfying (1) of Theorem 9 and (2) of Theorem 9 and

(1) {*F*, *f*} and {*G*, *g*} are *w*-compatible.
(2) Suppose that either
  (a) *g*(*X*) is a closed subset of *X* and *G*(*X* × *X*)⊆*f*(*X*) or
  (b) *f*(*X*) is a closed subset of *X* and *F*(*X* × *X*)⊆*g*(*X*).

Then *F*, *G*, *f*, and  *g* have a common coupled fixed point.

**Proof**

Since {*F*, *f*} and {*G*, *g*} satisfy the common (EA) property, there exist sequences {*x*_*n*_}, {*y*_*n*_}, {*u*_*n*_}, and {*v*_*n*_} in *X*, some *u*, *v* in *X*, and *A*, *B*, *C*, *D* in CB(*X*) satisfying (9). Suppose (a) holds; that is, *g*(*X*) is a closed subset of *X*, and then there exist $\tilde{x},\tilde{y}\in X$, and we have

(24) $$\begin{array}{c} u=g\tilde{x},\;\;v=g\tilde{y}. \end{array}$$

As in Theorem 9, we can prove that

(25) $$\begin{array}{c} g\tilde{x}\in G\left(\tilde{x},\tilde{y}\right),\;\;g\tilde{y}\in G\left(\tilde{y},\tilde{x}\right). \end{array}$$

That is, $\left(\tilde{x},\tilde{y}\right)$ is a coupled coincidence point of *G* and *g*. Hence, $\left(\tilde{x},\tilde{y})\in C\{G,g\right\}$. From *w*-compatibility of {*G*, *g*}, we have $gG(\tilde{x},\tilde{y})\subseteq G(g\tilde{x},g\tilde{y})$; hence, $g^{2}\tilde{x}\in G(g\tilde{x},g\tilde{y})$ and $g^{2}\tilde{y}\in G(g\tilde{y},g\tilde{x})$; that is, *gu* ∈ *G*(*u*, *v*) and *gv* ∈ *G*(*v*, *u*). Now, we shall show that *u* = *gu* and *v* = *gv*. Suppose, not. Then, by condition (2) of Theorem 9, we get

(26) φHFxn,yn,Gu,v+HFyn,xn,Gv,u2 ≤φdfxn,gu+dfyn,gv2  −ψdfxn,gu+dfyn,gv2.

Letting *n* → *∞* in the above inequality, by using (9) and (i_*φ*_), we obtain

(27) φHA,Gu,v+HC,Gv,u2 ≤φdu,gu+dv,gv2  −lim⁡n→∞ψdfxn,gu+dfyn,gv2.

Since *u* ∈ *A*, *v* ∈ *C*, *gu* ∈ *G*(*u*, *v*), and *gv* ∈ *G*(*v*, *u*), therefore, by (i_*ψ*_), we get

(28) φdu,gu+dv,gv2 ≤φHA,Gu,v+HC,Gv,u2 ≤φdu,gu+dv,gv2  −lim⁡n→∞ψdfxn,gu+dfyn,gv2 <φdu,gu+dv,gv2,

which is a contradiction. Thus, *u* = *gu* and *v* = *gv*. Hence, we have

(29) $$\begin{array}{c} u=gu\in G\left(u,v\right),\;\;v=gv\in G\left(v,u\right). \end{array}$$

Since *G*(*X* × *X*)⊆*f*(*X*), then there exist *x*, *y* ∈ *X* such that *fx* = *u* = *gu* ∈ *G*(*u*, *v*) and *fy* = *v* = *gv* ∈ *G*(*v*, *u*). Now, by condition (2) of Theorem 9, (i_*φ*_),  (ii_*φ*_), and (ii_*ψ*_), we get

(30) φDFx,y,u+DFy,x,v2 ≤φHFx,y,Gu,v+HFy,x,Gv,u2 ≤φdfx,gu+dfy,gv2  −ψdfx,gu+dfy,gv2 ≤0−0=0,

which, by (i_*φ*_) and (ii_*φ*_), implies

(31) $$\begin{array}{c} D\left(F\left(x,y\right),u\right)=D\left(F\left(y,x\right),v\right)=0. \end{array}$$

Thus,

(32) $$\begin{array}{c} u=fx\in F\left(x,y\right),\;\;v=fy\in F\left(y,x\right). \end{array}$$

That is, (*x*, *y*) is a coupled coincidence point of *F* and *f*. Hence, (*x*, *y*) ∈ *C*{*F*, *f*}. From *w*-compatibility of {*F*, *f*}, we have *fF*(*x*, *y*)⊆*F*(*fx*, *fy*); hence *f* ^2^ *x* ∈ *F*(*fx*, *fy*) and *f* ^2^ *y* ∈ *F*(*fy*, *fx*); that is, *fu* ∈ *F*(*u*, *v*) and *fv* ∈ *F*(*v*, *u*). Now, we shall show that *fu* = *u* and *fv* = *v*. Suppose, not. Then, by condition (2) of Theorem 9 and (ii_*ψ*_), we get

(33) φdfu,u+dfv,v2 ≤φHFu,v,Gu,v+HFv,u,Gv,u2 ≤φdfu,gu+dfv,gv2  −ψdfu,gu+dfv,gv2 ≤φdfu,u+dfv,v2  −ψdfu,u+dfv,v2 <φdfu,u+dfv,v2,

which is a contradiction. Thus, *fu* = *u* and *fv* = *v*. Hence, we have

(34) $$\begin{array}{c} u=fu\in F\left(u,v\right),\;\;v=fv\in F\left(v,u\right). \end{array}$$

Therefore, (*u*, *v*) is a common coupled fixed point of the pairs {*F*, *f*} and {*G*, *g*}. The proof is similar when (b) holds.

If we put *f* = *g* in Theorem 15, we get the following result.

**Corollary 16 .**

Let (*X*, *d*) be a metric space. Assume *F*, *G* : *X* × *X* → CB(*X*) and *g* : *X* → *X* to be mappings satisfying (1) of Corollary 10 and (2) of Corollary 10 and

(1) {*F*, *g*} and {*G*, *g*} are *w*-compatible;
(2) suppose that either
  (a) *g*(*X*) is a closed subset of *X* and *G*(*X* × *X*)⊆*g*(*X*) or
  (b) *g*(*X*) is a closed subset of *X* and *F*(*X* × *X*)⊆*g*(*X*).

Then *F*, *G*, and  *g* have a common coupled fixed point.

If we put *F* = *G* and *f* = *g* in Theorem 15, we get the following result.

**Corollary 17 .**

Let (*X*, *d*) be a metric space. Assume *F* : *X* × *X* → CB(*X*) and *g* : *X* → *X* to be mappings satisfying (1) of Corollary 11 and (2) of Corollary 11 and

(1) {*F*, *g*} is *w*-compatible;
(2) *g*(*X*) is a closed subset of *X* and *F*(*X* × *X*)⊆*g*(*X*).

Then *F* and *g* have a common coupled fixed point.

**Corollary 18 .**

Let (*X*, *d*) be a metric space. Assume *F*, *G* : *X* × *X* → CB(*X*) and *f*, *g* : *X* → *X* to be mappings satisfying (1) of Theorem 9, (1) of Corollary 12, (1) of Theorem 15, and (2) of Theorem 15; then *F*, *G*, *f*,  and *g* have a common coupled fixed point.

**Proof**

If *ψ* ∈ Ψ, then for all *r* > 0, *rψ* ∈ Ψ. If we divide condition (1) of Corollary 12 by 4 and take *φ*(*t*) = (1/2)*t*, *t* ∈ [0, +*∞*), then it reduces to condition (2) of Theorem 9 with *ψ*_1_ = (1/2)*ψ* and hence by Theorem 15 we get Corollary 18.

If we put *f* = *g* in Corollary 18, we get the following result.

**Corollary 19 .**

Let (*X*, *d*) be a metric space. Assume *F*, *G* : *X* × *X* → CB(*X*) and *g* : *X* → *X* to be mappings satisfying (1) of Corollary 10, (1) of Corollary 13, (1) of Corollary 16, and (2) of Corollary 16; then *F*, *G*, and *g* have a common coupled fixed point.

If we put *F* = *G* and *f* = *g* in Corollary 18, we get the following result.

**Corollary 20 .**

Let (*X*, *d*) be a metric space. Assume *F* : *X* × *X* → CB(*X*) and *g* : *X* → *X* to be mappings satisfying (1) of Corollary 11, (1) of Corollary 14, (1) of Corollary 17, and (2) of Corollary 17; then *F* and *g* have a common coupled fixed point.

**Theorem 21 .**

Let (*X*, *d*) be a metric space. Assume *F*, *G* : *X* × *X* → CB(*X*) and *f*, *g* : *X* → *X* to be mappings satisfying (2) of Theorem 9 and

(1) {*F*, *f*} and {*G*, *g*} are occasionally *w*-compatible.

Then *F*, *G*, *f*,  and *g* have a common coupled fixed point.

**Proof**

Since the pairs {*F*, *f*} and {*G*, *g*} are occasionally *w*-compatible, therefore there exist some points (*x*, *y*),$(\tilde{x},\tilde{y})\in X\times X$, such that

(35) $$\begin{array}{c} fx\in F\left(x,y\right),\;fy\in F\left(y,x\right),\;fF\left(x,y\right)\subseteq F\left(fx,fy\right),\\ g\tilde{x}\in G\left(\tilde{x},\tilde{y}\right),\;g\tilde{y}\in G\left(\tilde{y},\tilde{x}\right),\;gG\left(\tilde{x},\tilde{y}\right)\subseteq G\left(g\tilde{x},g\tilde{y}\right). \end{array}$$

It follows that

(36) $$\begin{array}{c} f^{2}x\in F\left(fx,fy\right),\;f^{2}y\in F\left(fy,fx\right),\\ g^{2}\tilde{x}\in G\left(g\tilde{x},g\tilde{y}\right),\;g^{2}\tilde{y}\in G\left(g\tilde{y},g\tilde{x}\right). \end{array}$$

Now, we shall show that $u=fx=g\tilde{x}$ and $v=fy=g\tilde{y}$. Suppose, not. Then, by condition (2) of Theorem 9 and (ii_*ψ*_), we have

(37) φdfx,gx~+dfy,gy~2 ≤φHFx,y,Gx~,y~+HFy,x,Gy~,x~2 ≤φdfx,gx~+dfy,gy~2  −ψdfx,gx~+dfy,gy~2 <φdfx,gx~+dfy,gy~2,

which is a contradiction. Thus, $fx=g\tilde{x}$ and $fy=g\tilde{y}$. Hence,

(38) $$\begin{array}{c} u=fx=g\tilde{x},\;\;v=fy=g\tilde{y}. \end{array}$$

Thus, by (36), we get

(39) $$\begin{array}{c} fu\in F\left(u,v\right),\;fv\in F\left(v,u\right),\\ gu\in G\left(u,v\right),\;gv\in G\left(v,u\right). \end{array}$$

Now, we shall show that *u* = *fu* = *gu* and *v* = *fv* = *gv*. Suppose, not. Then, by condition (2) of Theorem 9 and (ii_*ψ*_), we have

(40) φdfu,u+dfv,v2 ≤φHFu,v,Gx~,y~+HFv,u,Gy~,x~2 ≤φdfu,gx~+dfv,gy~2  −ψdfu,gx~+dfv,gy~2 ≤φdfu,u+dfv,v2  −ψdfu,u+dfv,v2 <φdfu,u+dfv,v2,

which is a contradiction. Thus,

(41) $$\begin{array}{c} u=fu,\;\;v=fv. \end{array}$$

Similarly, we can show that

(42) $$\begin{array}{c} u=gu,\;\;v=gv. \end{array}$$

Thus, by (39), (41), and (42), we get

(43) $$\begin{array}{c} u=fu\in F\left(u,v\right),\;\;v=fv\in F\left(v,u\right),\\ u=gu\in G\left(u,v\right),\;\;v=gv\in G\left(v,u\right). \end{array}$$

That is, (*u*, *v*) is a common coupled fixed point of *F*, *G*, *f*,  and *g*.

Put *f* = *g* in Theorem 21, and we get the following result.

**Corollary 22 .**

Let (*X*, *d*) be a metric space. Assume *F*, *G* : *X* × *X* → CB(*X*) and *g* : *X* → *X* to be mappings satisfying (2) of Corollary 10 and

(1) {*F*, *g*} and {*G*, *g*} are occasionally *w*-compatible.

Then *F*, *G*,  and *g* have a common coupled fixed point.

Put *F* = *G* and *f* = *g* in Theorem 21, and we get the following result.

**Corollary 23 .**

Let (*X*, *d*) be a metric space. Assume *F* : *X* × *X* → CB(*X*) and *g* : *X* → *X* to be mappings satisfying (2) of Corollary 11 and

(1) {*F*, *g*} is occasionally *w*-compatible.

Then *F* and *g* have a common coupled fixed point.

**Corollary 24 .**

Let (*X*, *d*) be a metric space. Assume *F*, *G* : *X* × *X* → CB(*X*) and *f*, *g* : *X* → *X* to be mappings satisfying (1) of Corollary 12 and (1) of Theorem 21; then *F*, *G*, *f*, and  *g* have a common coupled fixed point.

**Proof**

If *ψ* ∈ Ψ, then for all *r* > 0, *rψ* ∈ Ψ. If we divide condition (1) of Corollary 12 by 4 and take *φ*(*t*) = (1/2)*t*, *t* ∈ [0, +*∞*), then it reduces to condition (2) of Theorem 9 with *ψ*_1_ = (1/2)*ψ* and hence by Theorem 21 we get Corollary 24.

Put *f* = *g* in Corollary 24, and we get the following result.

**Corollary 25 .**

Let (*X*, *d*) be a metric space. Assume *F*, *G* : *X* × *X* → CB(*X*) and *g* : *X* → *X* to be mappings satisfying (1) of Corollary 13 and (1) of Corollary 22; then *F*, *G*, and *g* have a common coupled fixed point.

Put *F* = *G* and *f* = *g* in Corollary 24, and we get the following result.

**Corollary 26 .**

Let (*X*, *d*) be a metric space. Assume *F* : *X* × *X* → CB(*X*) and *g* : *X* → *X* to be mappings satisfying (1) of Corollary 14 and (1) of Corollary 23; then *F* and *g* have a common coupled fixed point.

**Example 27 .**

Suppose that *X* = [0,1], equipped with the metric *d* : *X* × *X* → [0, +*∞*) defined as *d*(*x*, *y*) = max⁡{*x*, *y*} and *d*(*x*, *x*) = 0 for all *x*, *y* ∈ *X*. Let *F*, *G* : *X* × *X* → CB(*X*) be defined as

(44) $$\begin{array}{c} F\left(x,y\right)=\left\{\begin{array}{cc} \left\{0\right\}, & \text{for}\;\;x,y=1\\ \left[0,\frac{x^{2}+y^{2}}{4}\right], & \text{for}\;\;x,y\in \left[0,1\right), \end{array}\right.\\ G\left(x,y\right)=\left\{\begin{array}{cc} \left\{0\right\}, & \text{for}\;\;x,y=1\\ \left[0,\frac{x+y}{8}\right], & \text{for}\;\;x,y\in \left[0,1\right). \end{array}\right. \end{array}$$

Suppose *f*, *g* : *X* → *X* be defined as

(45) $$\begin{array}{c} fx=\left\{\begin{array}{cc} x^{2}, & x\neq 1,\\ \frac{3}{2}, & x=1, \end{array}\right.\;\forall x\in X,\\ gx=\left\{\begin{array}{cc} \frac{x}{2}, & x\neq 1,\\ 1, & x=1, \end{array}\right.\;\forall x\in X. \end{array}$$

Define *φ* : [0, +*∞*)→[0, +*∞*) by

(46) $$\begin{array}{c} \varphi \left(t\right)=\frac{t}{2},\;\forall t>0, \end{array}$$

and *ψ* : [0, +*∞*)→[0, +*∞*) by

(47) $$\begin{array}{c} \psi \left(t\right)=\left\{\begin{array}{cc} \frac{t}{4}, & \text{for}\;\;t\neq 1\\ 1, & \text{for}\;\;t=1. \end{array}\right. \end{array}$$

Now, for all *x*, *y*, *u*, *v* ∈ *X* with *x*, *y*, *u*, *v* ∈ [0,1), we have

*Case *(*a)*. If (*x* ^2^ + *y* ^2^)/4 = (*u* + *v*)/8, then

(48) φHFx,y,Gu,v+HFy,x,Gv,u2 =14HFx,y,Gu,v+HFy,x,Gv,u =14u+v8+v+u8 ≤18max⁡x2,u2+max⁡y2,v2 ≤18dfx,gu+dfy,gv ≤14dfx,gu+dfy,gv2 ≤φdfx,gu+dfy,gv2  −ψdfx,gu+dfy,gv2.

*Case (b)*. If (*x* ^2^ + *y* ^2^)/4 ≠ (*u* + *v*)/8 with (*x* ^2^ + *y* ^2^)/4 < (*u* + *v*)/8, then

(49) φHFx,y,Gu,v+HFy,x,Gv,u2 =14HFx,y,Gu,v+HFy,x,Gv,u =14u+v8+v+u8 ≤18max⁡x2,u2+max⁡y2,v2 ≤18dfx,gu+dfy,gv ≤14dfx,gu+dfy,gv2 ≤φdfx,gu+dfy,gv2  −ψdfx,gu+dfy,gv2.

Similarly, we obtain the same result for (*u* + *v*)/8<(*x* ^2^ + *y* ^2^)/4. Thus, the contractive condition (2) of Theorem 9 is satisfied for all *x*, *y*, *u*, *v* ∈ *X* with *x*, *y*, *u*, *v* ∈ [0,1). Again, for all *x*, *y*, *u*, *v* ∈ *X* with *x*, *y* ∈ [0,1) and *u*, *v* = 1, we have

(50) φHFx,y,Gu,v+HFy,x,Gv,u2 =14HFx,y,Gu,v+HFy,x,Gv,u =14x2+y24+y2+x24 ≤18max⁡x2,u2+max⁡y2,v2 ≤18dfx,gu+dfy,gv ≤14dfx,gu+dfy,gv2 ≤φdfx,gu+dfy,gv2  −ψdfx,gu+dfy,gv2.

Thus, the contractive condition (2) of Theorem 9 is satisfied for all *x*, *y*, *u*, *v* ∈ *X* with *x*, *y* ∈ [0,1) and *u*, *v* = 1. Similarly, we can see that the contractive condition (2) of Theorem 9 is satisfied for all *x*, *y*, *u*, *v* ∈ *X* with *x*, *y*, *u*, *v* = 1. Hence, the hybrid pairs {*F*, *f*} and {*G*, *g*} satisfy condition (2) of Theorem 9, for all *x*, *y*, *u*, *v* ∈ *X*. In addition, all the other conditions of Theorem 9, Theorem 15, and Theorem 21 are satisfied and *z* = (0,0) is a common coupled fixed point of *F*, *G*, *f*, and  *g*.
